# Platelet serotonin level and its correlation with finger length relation

**DOI:** 10.4103/0019-5545.44746

**Published:** 2008

**Authors:** Devasis Ghosh

**Affiliations:** Department of Psychiatry, Rehmatbai Hospital, 82A Raja Ram Mohan Roy Road, Kolkata - 700 082, India

**Keywords:** Finger lengths (4D and 5D), neuroticism, personality, serotonin

## Abstract

**Background:**

Serotonin is one of the neurotransmitters implicated in normal personality. Many psychobiological models of personality include some dimensions related to serotonin. Moreover, platelets have long been proposed as easily obtainable, neurological models of serotonergic neurons.

**Aim:**

This study was done to examine whether measurement for platelet serotonin could aid as a marker for increased neuroticism trait amongst (arbitrarily named Group C) males and females having the tip of the little fingers below the distal finger mark on the adjacent ring fingers in both their outstretched hands compared to (arbitrarily named Group A) the males and females who have the tip of the little fingers above the distal finger mark on the adjacent ring fingers in both their outstretched hands.

**Materials and Methods:**

Platelet serotonin estimation was done by Elisa Method (LDN, Germany Kit) from randomly selected 48 healthy subjects [24 males (12 males belonging to Group A and 12 belonging to Group C) and 24 females (12 females belonging to group A and 12 females belonging to Group C)].

**Results:**

Preliminary results showed that the platelet serotonin levels were significantly lower (level of significance: 0.05 in *t*-Test analysis) in Group C males compared to those observed in Group A males and the platelet serotonin levels were also significantly lower (level of significance: 0.05 in *t*-Test analysis) in Group C females compared to those observed in Group A females.

**Conclusion:**

Thus, it may be inferred that the platelet serotonin may be used as a biochemical marker for increased neuroticism trait in Group C subjects.

## INTRODUCTION

Serotonin is one of the neurotransmitters implicated in normal personality. Many psychobiological models of personality include some dimensions related to serotonin.

It has got extensive effects on “some fundamental aspects of physiology and behavior, ranging from the control of body temperature, cardiovascular activity and respiration to involvement in such behaviors as aggression, eating, and sleeping‘.[1]

The current biological theory of depression is based on evidence that biogenic amines are involved in mood regulation.[2,3] “Heterogeneous dysregularities” in one or more amines are implied, generally involving excesses of amines in mania and deficiencies of amines in depression.[3]

Jacobs[1] draws connections from serotonin function to depression and compulsions. Besides playing major roles in mood and compulsions, serotonin has also been implicated in other complex behaviors.

Low serotonin levels have long been associated with impulsive behavior (e.g., fire-setting), in nonparanoid schizophrenia, nonpsychotic depression and suicidal behavior[3–5] and with a predisposition to violence.[6,7] Maurer-Spurej and colleagues[8] also showed reduced platelet serotonin levels in patients with depression. It was suggested by Muck-Seler[9] that even the differences in platelet 5-HT concentrations found amongst depressed patients may be used as a biological marker for suicidal behavior i.e., lowest platelet 5-HT concentrations were associated with the most pronounced suicidal behavior.

Also Marazzitti, *et al.*[10] found that highly aggressive individuals had reduced levels of serotonin when compared with controls. Raine's[11] research showed that groups of antisocial individuals display lowered central serotonin levels and he concluded therefore that serotonin is involved in decreasing aggression. Goveasa *et al*.[12] showed an association between reduced platelet 5-HT content and aggression in personality disorder (PD) subjects. Platelet serotonin levels were found to be decreased during treatment with recombinant interferon leading to increased irritability.[13]

Higher platelet serotonin content was found to be associated with co morbid borderline personality disorder and behavior traits such as aggressivity[5] and in patients with psychotic features (i.e., in paranoid schizophrenia and psychotic depression).[4] Wright[14] had even suggested that serotonin may “regulate self-esteem in accordance with social feedback” and thus, the level of serotonin in the brain may be both genetically set within some given range and may also be modulated by social experience. A significant increase in platelet 5-HT levels in bipolar depressives compared to normal controls was found in a study by Shiah *et al*.[15] Findings by Neuger *et al*.[16] indicates that both the temperament and character dimensions may be serotonergically modulated.

Neurons and platelets display structural and functional similarities and moreover in blood more than 99% of 5-HT is contained in platelets so that the platelets have been proposed as an ideal peripheral model of central functions.[17]

Platelets also share a similar serotonin uptake and release mechanism with serotonergic neurons.[18–20] In addition, peripheral and brain serotonin might be linked, because the blood-brain barrier seems to act as a regulatory interface for neurotransmitters. Especially under stressful conditions, circulating serotonin is able to cross the blood-brain barrier in humans and experimental animals.[21–23] It has also been clearly shown by Bianchi *et al*.[24] that changes in 5-HT levels in platelets corresponds with the changes in central or neuronal 5-HT.

Because of its ease of use, platelet serotonin measurements could be performed routinely to support the results obtained with questionnaires. Though the platelet serotonin level does not show a circadian rhythm[25,26] yet a circadian rhythm of platelet serotonin uptake and release exists.[27] Moreover no significant correlations between platelet serotonin levels and sex or age have been found in healthy donors.[28,29] This independence of platelet serotonin levels from external parameters is thus advantageous for comparative studies and for general use as a clinical marker.

The study by Ghosh[30] showed that both males and females having the tip of the little fingers below the distal finger mark on the adjacent ring fingers in both their outstretched hands (arbitrarily named Group C) will have higher Neuroticism scores (i.e., they will be more anxious, worried, moody, unstable and be easily upset in the face of very minor stresses), compared to the males and females who have the tip of the little fingers above the distal finger mark on the adjacent ring fingers in both their outstretched hands (arbitrarily named Group A).

This characteristic personality trait in Group C people may hence act as a predisposing factor in the development of affective disorders, neurotic disorders, deliberate self harm (DSH), and substance abuse.

The overall hypothesis for this study was that platelet serotonin could aid as a biochemical marker for increased/high neuroticism trait and support the results from EPQ questionnaires amongst (arbitrarily named Group C) males and females having the tip of the little fingers below the distal finger mark on the adjacent ring fingers in both their outstretched hands compared to (arbitrarily named Group A) the males and females who have the tip of the little fingers above the distal finger mark on the adjacent ring fingers in both their outstretched hands. If such a correlation could be demonstrated, platelet serotonin measures could aid in determining personality trait of neuroticism.

## MATERIALS AND METHODS

Platelet serotonin estimation was done by Elisa Method (LDN, Germany Kit) from randomly selected 48 healthy subjects belonging to the city of Kolkata. Amongst the volunteers there were 24 males (12 males belonging to Group A and 12 belonging to Group C) and 24 females (12 females belonging to Group A and 12 females belonging to Group C). The average age of volunteers ranged from 19 to 54 yrs (mean age: 34.2 yrs). The volunteers had participated in the study after providing written informed consent and confirmed that they had no medical condition and had not taken any medication for at least 2 weeks before blood donation.

Diet restrictions which might affect serotonin level were followed for 24 hrs before testing. EPQ-R Questionnaire was given to the volunteers after blood collection.

The standard protocol as specified in the kit manual was followed: Initially PRP (Platelet rich plasma) was obtained by centrifugation of the collected blood sample in EDTA vial for 10 min at room temperature at 200 *g*. Platelet pellets were then obtained by adding 800 μl of physiological saline to 200 μl of the supernatant of PRP and centrifuged for 10 min at 4°C at 4,500*g*. after discarding the supernatant 200 μl of distilled water is added the pellets and mixed thoroughly in a vortex mixture. 10 μl of the supernatant was used for acylation reaction. Serotonin antiserum was added to 10 μl of acylated solution, washed thoroughly, enzyme conjugate, then substrate and then stop solution were added successively. The absorbance of the solution in the wells was read within 10 min using a microplate reader set to 450 nm.

### Statistical analysis

*t*-test analysis was used to determine whether mean platelet serotonin levels as determined by Elisa were significantly different in healthy Group A males and females subjects, compared with healthy Group C males and females subjects.

## RESULTS

### Platelet serotonin levels

**Figure 1:**
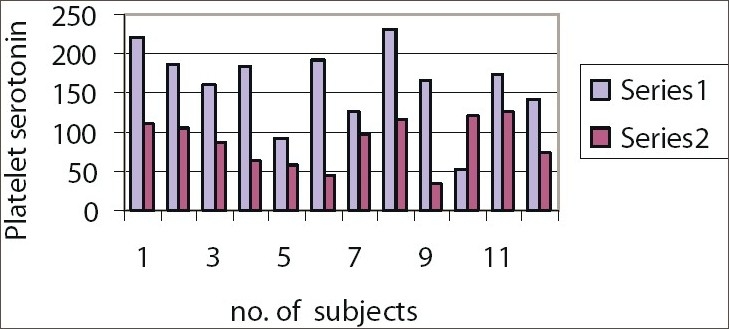
Platelet serotonin levels in males. Series 1: Group A Males, Series 2: Group C Males

**Figure 2:**
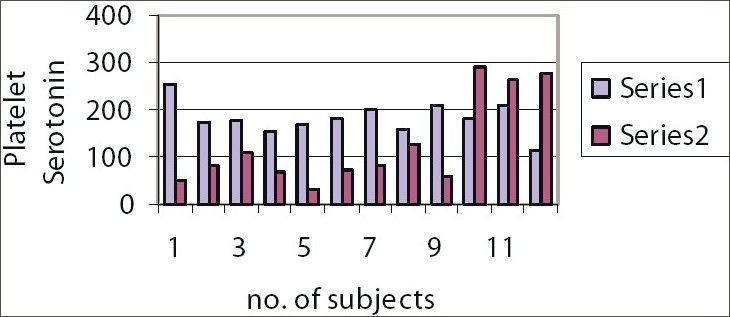
Platelet serotonin levels in females. Series 1: Group A Females, Series 2: Group C Females

vide Table 1 and Figure 1 and Figure 2, *t-* test (Paired Two Sample for Means) analysis showed that

**Table 1:** Platelet serotonin levels

No.	Male A	Male C	No.	Female A	Female C
1	220.23	110.37	1	252.87	48.16
2	187.43	105.4	2	174.93	81.83
3	159.53	87.67	3	179	110.37
4	183.17	63.5	4	155.9	68.06
5	91.8	57.93	5	167.07	31.83
6	191.8	46	6	183.6	72.93
7	126.73	96.13	7	200.86	83.73
8	230.6	115.57	8	159.53	126.73
9	167.06	33.3	9	210.33	57.93
10	51.36	121.03	10	183.17	290.33
11	174.93	126.73	11	210.33	264.77
12	142.2	72.93	12	115.57	277.27
Mean	160.57	86.38	Mean	182.7633	126.1617
SD	51.39791	31.15451	SD	34.19372	94.80141

*t*-test: Paired two sample for means, Level of significance: 0.05, df: 11, *t* Critical two-tail: 2.200986273

1. Mean platelet serotonin level was significantly low (level of significance= 0.05) in healthy Group C males subjects, compared with healthy Group A males subjects (df=11, *t* Critical two-tail= 2.200986273)
2. Mean platelet serotonin level was significantly low (level of significance= 0.05) in healthy Group C females subjects, compared with healthy Group A females subjects (df=11, *t* Critical two-tail= 2.200986273).

## DISCUSSION

This study shows that

1. Amongst the males, the platelet serotonin level is significantly low in Group C males compared to Group A males. Therefore, lower platelet serotonin level which may account for the higher neuroticism scores in Group C males.
2. Amongst the females, the platelet serotonin level is significantly low in Group C females compared to Group A females. Therefore, lower platelet serotonin level which may account for the higher neuroticism scores in Group C females.

N.B. In this study, only in 3 out of 12 Group C females, the platelet serotonin was quite high compared to Group A females.

Therefore we can infer from this study that the difference in platelet serotonin level amongst Group A and Group C persons may account for difference in their Neuroticism scores (i.e., Group C persons have a higher neuroticism score compared to Group A persons…[30]).

## CONCLUSIONS

It may be inferred from this study that the platelet serotonin may be used as a biochemical marker for the increased Neuroticism trait amongst Group C human beings (males and females having the tip of the little fingers below the distal finger mark on the adjacent ring fingers in both their outstretched hands) compared to Group A human beings (males and females who have the tip of the little fingers above the distal finger mark on the adjacent ring fingers in both their outstretched hands).

Further studies may be undertaken to find out a normal range for platelet serotonin correlating with very low/zero Neuroticism scores. Pretreatment platelet serotonin measurement may be used to determine the choice of medication and the proper dosage required and its clinical responsiveness.[31]
